# Analysis of Predictors of Myocardial Infarction in Trauma With Development of a Trauma Cardiac Risk Index

**DOI:** 10.7759/cureus.13153

**Published:** 2021-02-05

**Authors:** John T Culhane, Michelle A Mangold, Carl Freeman

**Affiliations:** 1 Surgery, Saint Louis University School of Medicine, Saint Louis, USA; 2 Trauma, Saint Louis University School of Medicine, Saint Louis, USA

**Keywords:** trauma, myocardial infarction, risk assessment, comorbidity, cardiac

## Abstract

Study objective: Trauma has historically been considered a disorder of the young and healthy, with a low risk of cardiac ischemia; hence most research on myocardial infarction in trauma has focused on direct cardiac damage from blunt chest trauma. However, the age and comorbidity of trauma patients are increasing, making the trauma population more vulnerable to myocardial infarction (MI). Cardiac risk assessment has emphasized morbidity and mortality in an elective surgical setting, but it is also important in acute trauma. Our study analyzes the risk factors for MI in a trauma population to create a scoring system to predict the risk of MI.

Design: Retrospective cohort analysis of a national trauma registry over a five-year period. Potential predictors of MI in trauma patients were identified and tested with univariate and multivariate statistics. A numerical score was created to predict the risk of MI based on these criteria.

Setting: The National Trauma Data Bank (NTDB) is a large registry of selected trauma centers in the United States. Data include demographic, injury, treatment, and outcome variables pertaining to the index admission of each patient. The institutions range from community hospitals through level 1 trauma centers. The time period is the entire inpatient hospital admission from arrival from the field, through the emergency department, ICU, and floor up to discharge.

Patients: 3,437,959 trauma patients aged 18 years and older from various US trauma centers. 62.8% were male. The median age is 50 years with a standard deviation of 21.25. The median Injury Severity Score is 9 with a standard deviation of 9.04.

Measurements: Demographic, traumatic, and comorbidity variables were collected from the NTDB. The primary outcome was MI during the initial trauma admission. Multivariate analysis was performed with logistic regression.

Main results: Over 8010 (0.23%) suffered an MI. The strongest risk factors for MI were a history of MI with an adjusted odds ratio (OR) of 7.0, and angina with an OR of 3.4. A procedure under general anesthesia (GA) conferred an OR of 2.3. Minor risk factors included torso injury and 10-year age interval over 50, both with an OR of 1.7, a 20-point interval of the Injury Severity Score (ISS) with OR 1.6, male gender with OR of 1.5, and various chronic disease comorbidities with OR ranging from 1.4 to 1.9. A Trauma Cardiac Risk Index (TCRI) was derived from these risk factors. The model showed good discrimination with a C statistic of 0.85.

Conclusions: Overall the trauma population has a low risk of MI. However, the risk is much higher for older patients with chronic comorbidity. The TCRI can be used to assess cardiac risk in trauma patients to help direct monitoring, testing, and risk reduction measures to those at the highest risk.

## Introduction

Trauma is typically regarded as a disorder of the young. Perhaps, for this reason, cardiac ischemia in trauma patients is not well studied. However, due to an aging demographic, the United States trauma population is becoming older, with greater chronic comorbidity [1]. Much of the literature regarding myocardial infarction (MI) in trauma discusses the effect of direct cardiac damage from chest trauma, but trauma patients can suffer from demand-related ischemic heart disease as well [2-4]. Trauma, like surgery, can cause physiologic stress which could potentially lead to cardiac ischemia and MI. There is a great deal of literature regarding risk stratification and reduction of peri-operative cardiac morbidity, but similar studies in the trauma population are lacking [5,6]. Identifying high-risk patients would allow increased surveillance and cardiac risk reduction for the more vulnerable patients. Conversely, low yield testing could be avoided for the group at minimal risk. The objective of this study is to develop a tool to calculate the risk of MI in the trauma population. This will allow clinicians to direct resources to the trauma patients at the greatest cardiac risk.

## Materials and methods

The National Trauma Data Bank (NTDB) is a registry of trauma data from multiple United States trauma centers. The years 2011 through 2015 were chosen. Patients 18 years and older were selected. Potential predictors of MI were identified. Types of variables analyzed were demographic, body region of injury, type of procedure during admission, and medical comorbidity. We selected procedures that were likely to require general anesthesia (GA), by examining the list of ICD9 procedure codes. Non-surgical and minor surgical procedures were excluded. Schwarze et al. identified a set of surgical procedures that were high risk for complications [7]. We cross-referenced this list of procedures with the procedure codes in the NTDB to identify high-risk procedures.

For univariate analysis of categorical variables, Chi-square was used to test the significance. Multivariate analysis was performed with logistic regression.

Risk factors that were statistically significant, with an adjusted odds ratio (OR) of greater than 1.4 were included in the final model. A risk score was created based on the adjusted OR. One point was given for OR of 1-3, two points for 3-6, and three points for 6 or greater. We called this the Trauma Cardiac Risk Index (TCRI).

For comparison, a risk score was calculated based on the Revised Cardiac Risk Index (RCRI), commonly used for perioperative risk stratification. [5] Cardiac ischemia was considered positive if the patient had a history of myocardial infarction or angina. A point was assigned for cardiac ischemia, cerebrovascular infarction, chronic renal failure, congestive heart failure, and diabetes.

We assessed the discriminative ability of the risk scores using a receiver operator curve (ROC). Higher values of area under the curve or C statistic indicate better discriminative ability. This ranges from 0.5, which indicates a random chance to 1.0, which indicates perfect discrimination. C statistic was calculated for both the TCRI and the RCRI.

Statistics were performed with the SPSS statistical package. SPSS version 24.0 (IBM Corp., Armonk, NY, USA).

## Results

Baseline characteristics

Over 3,437,959 patients were included in the analysis; 62.8% are male and 37.2% are female. The mean age is 50.81. The median age is 50 years with a standard deviation of 21.25. The mean ISS is 10.15. The median ISS is 9 with a standard deviation of 9.04. 30.0% of MI’s were associated with a chest injury. Mortality for all patients and for the subgroup who did not suffer an MI is identical at 2.8%. Mortality is 22.6% for those who did suffer an MI (p<0.001). Seven hundred and eighteen patients suffered a direct traumatic cardiac injury. The majority, 457 (63.6%) were cardiac contusions without open wound into the thorax. Only three of the 718 direct cardiac injury patients suffered an MI. Types of major surgery are listed in Table 1.

**Table 1:** Major types of procedures by organ system. Musculoskeletal, including major orthopedic procedures, are classified as intermediate cardiac risk [6,7]. GA: general anesthesia.

Type of Procedure	All Under GA		All High Risk	
	n	%	n	%
Nervous system	266,270	7.36	90868	42.83
Endocrine	4584	0.13	120	0.06
Eye	34,132	0.94	0	0
Nose, mouth, pharynx	50,270	1.39	0	0
Respiratory	173,811	4.81	4236	2
Cardiovascular	165,519	4.58	36047	16.99
Digestive	365,259	10.1	72513	34.18
Urinary	27,149	0.75	8217	3.87
Musculoskeletal	2,430,788	67.2	153	0.07

Females had a slightly higher incidence of MI than males. The incidence of MI rose steadily with increasing age. Eight chronic medical conditions typically associated with atherosclerosis were associated with an increased risk of MI. The risk of MI was higher for patients who underwent any procedure under GA, and higher still if the procedure was high risk. The risk of MI increased with increasing ISS, except for the very highest scores. The particular body region injured was not a major risk factor for MI. The chest and lower extremities were the body regions with the greatest increased risk (Table 2).

**Table 2:** Unadjusted risk factors for myocardial infarction MI: myocardial infarction.

	No MI	MI	%	p-Value
Demographics				
Gender				
Male	2,153,946	4915	0.23	0.008
Female	1,276,003	3095	0.24	
Age (years)				
18-49	1,672,124	591	0.04	<0.001
50-59	520,337	960	0.18	<0.001
60-69	421,599	1577	0.37	<0.001
70-79	376,756	2148	0.57	<0.001
80+	439,133	2734	0.62	<0.001
Comorbidity				
Angina	5844	226	3.72	<0.001
History of MI	41,595	1504	3.49	<0.001
Peripheral vascular disease	15,068	228	1.49	<0.001
Congestive heart failure	105,854	1324	1.24	<0.001
Chronic renal failure	33,146	319	0.95	<0.001
Cerebrovascular accident	75,381	662	0.87	<0.001
Diabetes	491,919	2559	0.52	<0.001
Hypertension	987,818	5157	0.52	<0.001
Procedures				
Procedure under general anesthesia	1,334,584	4736	0.35	<0.001
High-risk procedure	147,356	857	0.58	<0.001
Injury				
Injury severity score				
0-19	2,976,840	6052	0.20	<0.001
20-39	383,648	1706	0.44	<0.001
40-59	33,587	192	0.57	<0.001
60+	8505	30	0.35	0.02
Body region injured				
Chest	808,763	2400	0.30	<0.001
Lower extremity	1,333,824	3985	0.30	<0.001
Abdomen	404,642	1145	0.28	<0.001
Spine	637,558	1774	0.28	<0.001
Head	1,230,462	3212	0.26	<0.001
Upper extremity	1,021,125	2257	0.22	0.002
Face	849,095	1757	0.21	<0.001
Neck	72,779	140	0.19	0.02
Total	3,429,949	8010	0.23	

In the multivariate analysis, previous cardiac ischemia, consisting of a history of MI and angina, were the strongest risk factors for MI. Any procedure under GA doubled the OR. After the top three risk factors, various medical comorbidities, demographic characteristics, and injury patterns conferred moderately increased risk (Table 3).

**Table 3:** Multivariate analysis of risk factors for myocardial infarction GA: general anesthesia, MI: myocardial infarction, OR: odds ratio.

Risk Factor	Adjusted OR	p-Value
History of MI	7.03	<0.001
Angina	3.41	<0.001
Procedure under GA	2.28	<0.001
Congestive heart failure	1.87	<0.001
Peripheral vascular disease	1.70	<0.001
Age interval	1.68	<0.001
Hypertension	1.65	<0.001
Torso injury	1.65	<0.001
Injury severity score interval	1.63	<0.001
Diabetes	1.56	<0.001
Male gender	1.48	<0.001
High-risk procedure	1.47	<0.001
Cerebrovascular accident	1.43	<0.001
Chronic renal failure	1.42	<0.001

**Figure 1:**
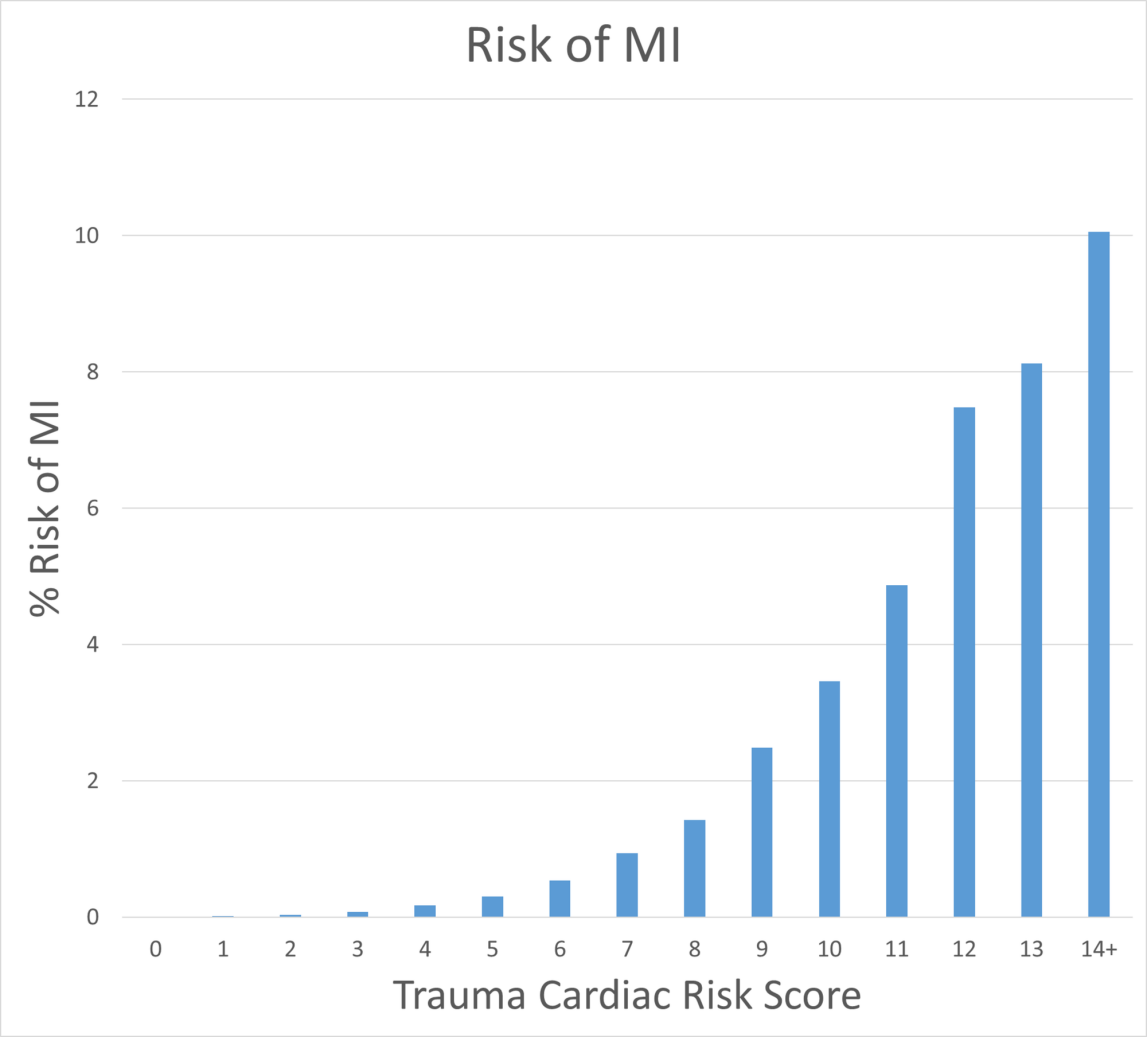
Risk of MI for each Trauma Cardiac Risk Score MI: myocardial infarction.

The risk of MI increases with a higher Trauma Cardiac Risk Score, calculated based on the multivariate analysis. The risk is virtually zero with a lower score, ranging up to 10% risk with the highest. The ROC for the prediction of MI shows the good discriminative ability of the model. The C Statistic for the TCRI is 0.85. For comparison, the C Statistic using the RCRI to predict MI in this trauma dataset is 0.72 (Figure 1).

## Discussion

Trauma has traditionally been considered a disease of the young, but the average age of the trauma population has been increasing year by year. From the years 2000 to 2011, the mean age of trauma inpatients in the US increased from 54.08 to 59.58 years [1]. With increasing age comes increasing chronic comorbidity. Over the same period, the proportion of trauma inpatients with a Charlson Comorbidity Index score greater than or equal to 3 increased from 0.048 to 0.139, an increase of nearly threefold [1]. In our study, we found that the comorbidities typically associated with cardiovascular risk were universally harmful. They are all associated with a higher risk of MI. Thus, with an older, sicker trauma population, we can expect to see more cardiac complications.

Trauma has previously been identified as a risk factor for MI [8]. Many studies describe MI as a direct result of cardiac injury due to trauma, most often blunt trauma. This often involves traumatic dissection of coronary arteries [2-4]. However, MI in trauma is not always associated with direct damage to the coronary arteries or myocardium. Many cases show no coronary artery damage [4]. Others do not involve chest trauma at all. Ismailov et al. described predictors of MI in trauma. Blunt cardiac injury (BCI) was associated with a 2.6-fold increase in acute MI, but abdominal and pelvic trauma was also associated with a 65% increase [8]. These results show that non-thoracic trauma can also increase the risk of MI. In our study, most MI’s were not due to direct cardiac trauma. Only 30.0% were associated with any chest injury at all. In the multivariate analysis, chest trauma was a significant, but a minor predictor of MI.

Most MI’s in this study was due to a mechanism other than direct damage to the heart. In the general population, as well as in trauma, MI may occur without direct coronary artery damage or obstructive coronary atherosclerosis. Type 2 myocardial infarction (supply/demand mismatch) occurs when increased myocardial oxygen demand outstrips available supply. This leads to myocardial injury in the absence of direct trauma or ruptured atherosclerotic plaque [9]. In trauma, the reduced supply may be due to hypoxemia, anemia, and hypotension. The increased demand may be due to tachyarrhythmia and increased metabolic rate due to systemic inflammation. In an analysis of provoking factors of type 2 MI, Smilowitz and Tsafrir identified several conditions that are frequently seen in severe acute trauma: anemia or bleeding requiring transfusion (32%), tachyarrhythmia (23%), hypotension (22%), and respiratory failure (23%) [10]. Acute emotional stress, such as that may occur in trauma, is also a risk factor for MI and sudden death [11].

The risk of MI due to the physiologic stress of trauma is not well studied, but there is extensive literature related to an analogous situation. Non-cardiac surgery presents cardiac risks that are similar to trauma. Surgery like trauma causes tissue damage, a hyperadrenergic state, and systemic inflammation with associated hypermetabolism. Both surgery and trauma may result in increased myocardial oxygen demand due to hemodynamic effects. They are both examples of physiologic stress that can provoke cardiac ischemia. A study of 215,077 non-cardiac surgery patients showed an overall incidence of peri-operative MI of 0.07%. Most of these were associated with comorbidity. Fifty percent had a diagnosis of coronary artery disease and 19% had heart failure; 72.6% was type 2 MI and 25.3 was type 1 [12]. Our overall incidence of MI was 0.23%, an incidence over three times higher than this non-cardiac surgery population.

In 1999, Lee et al. developed the RCRI to predict the risk of major cardiac events in the peri-operative setting [5]. This scoring system is still widely used to predict peri-operative cardiac risk. Major cardiac events were defined as MI, pulmonary edema, ventricular fibrillation or primary cardiac arrest, and complete heart block. The risk factors which make up the RCRI are coronary heart disease, congestive heart failure, cerebrovascular disease, insulin-dependent diabetes mellitus, renal insufficiency with serum creatinine measuring greater than 2 mg/dL, and high-risk surgery (suprainguinal vascular, intraperitoneal, or intrathoracic).

The RCRI model was validated with a C statistic of 0.77. We chose to analyze the risk of MI alone rather than a composite of cardiac events because, in a multi-system trauma, the other events might occur for various reasons other than cardiac. In our analysis, the RCRI performed almost as well in the trauma setting as in the peri-operative, predicting MI with a C statistic of 0.72. This shows that conventional peri-operative risk factors effectively identify trauma patients at high risk for MI. The TCRI, which we developed specifically for trauma, shows better discriminative ability than the RCRI. With a C statistic of 0.85, the TCRI outperformed the RCRI in the trauma setting and even performed better for trauma than the RCRI did for peri-operative patients.

Our study showed that a history of MI, with an OR of 7.0, was by far the strongest predictor of a patient suffering another MI during the trauma admission. A large database study from California showed that a history of recent MI was a strong risk factor for post-operative MI and mortality. The risk diminished with the time from MI to surgery [13]. Trauma can happen at any time interval after an MI, and for those patients who require surgery, it is not always possible to delay the procedure. This could account for some of the high risks of previous MI. Angina was the second strongest risk factor, with an OR of 3.4. Angina and previous MI represent ischemic heart disease. A study from 1990 showed that angina and history of MI greatly increased the risk of peri-operative MI [14]. Therefore, it is not surprising that patients with pre-existing coronary artery disease suffer the highest risk of subsequent MI [15]. Although MI due to direct cardiac trauma has received the most attention in the literature, chest trauma is not the strongest predictor of MI. Abdominal trauma predicts MI as well. In our study, any part of the torso, not only the chest, was the anatomic region associated with the highest risk. Injury to a more central anatomic region may provoke the greatest physiologic stress. ISS was a moderate predictor of the risk of MI. ISS is designed to predict mortality, but an anatomic injury scale does not necessarily represent the greatest physiologic derangement resulting in the greatest cardiac stress [16]. Older age and male sex are risk factors for MI in trauma as they are in the general population [17].

Practical application

Cardiac risk stratification is an important part of trauma care. Identifying high-risk patients allows increased surveillance such as admission to the intensive care unit and telemetry. For higher-risk patients, providers could consider non-surgical versus surgical treatment of injury where both are reasonable therapeutic alternatives. This could potentially reduce risk because having a procedure under GA was the third highest risk factor for MI with an OR of 2.3. Hypertension could be more strictly controlled and hemodynamics more closely monitored. The clinician could consider the judicious use of B blockers and statins.

Better risk stratification could help define the role of troponins in trauma. Elevated troponins are an essential part of the diagnosis of MI. [9]. Eastern Trauma Association Guidelines recommend measurement of cardiac troponins in patients suspected to have suffered BCI [18]. However, there is less consensus on the role of cardiac biomarkers in patients without blunt chest trauma. Troponins should be tested in patients with typical ischemic chest pain, but MI may occur without cardiac symptoms. In one peri-operative study, patients presented with precordial pain only 9% of the time [19]. In trauma, ischemic chest pain may be masked by chest wall injury, or in severe trauma the patient may not be able to communicate symptoms due to intubation. The decision to work up MI in trauma may depend significantly on a priori risk rather than symptoms.

Troponins have been shown to be useful for prognosis after trauma [20], but they are not highly specific for MI. Troponins are specific for injury to cardiac rather than skeletal muscle, but they often represent myocardial injury rather than type 1 MI. Elevated troponins are related to many non-cardiac conditions such as hypotension, traumatic brain injury, or hypoxemia [21,22]. A review of the role of cardiac troponin testing in elderly patients with hip fractures showed elevated troponins in 26.7 to 39% of patients. Of the troponin positive patients, myocardial infarction, cardiac complications, and cardiac death occurred in ≤35% [23]. In many emergency departments, cardiac troponins are used for screening even in the absence of symptoms of cardiac ischemia at a rate as high as one in five patients. This leads to a positive predictive value as low as 16.4% [24]. While troponins are biochemically specific, they are only clinically specific when ordered in patients for whom ischemic heart disease is plausible. When troponins are ordered indiscriminately, there will be more false positives with subsequent workup and possible harm from overtreatment [22]. An objective a priori risk assessment using the TCRI could be used to help decide when to order troponins and how to interpret the results.

Using the TCRI to guide monitoring and appropriate testing with troponins could potentially diagnose MI sooner. Therapeutic options are often limited in trauma. Thrombolytics are rarely permissible, but often aspirin or heparin can be given. In appropriate candidates, invasive treatment has led to better outcomes [4]. Conversely, the TCRI could be used to identify a low-risk subset of trauma patients who do not require screening for cardiac ischemia. The risk of major adverse cardiac events for non-cardiac surgery is divided into low-risk, intermediate-risk, and high-risk groups with estimated 30-day cardiac event rates (cardiac death and MI) of <1%, 1-5%, and >5%, respectively [6]. In our study, the low, intermediate, and high groups correspond to TCRI of 0-7, 8-11, and 9 and above, respectively. The vast majority (97%) are low risk. If they were being evaluated for non-cardiac surgery, they would need no further workup.

Strengths of study

We developed a simple score to quantify the risk of MI in trauma with readily available information. The score should be easy to implement in daily practice. The analysis has high statistical power. Over 3 million patients were included.

Weaknesses of study

Lack of gold standard for MI in trauma. It is possible that some of the reported MI’s could be cardiac injury [25]. The NTDB does not distinguish between STEMI and NSTEMI or specify the morbidity and mortality attributable to MI. Previous functional capacity is an important part of cardiac risk stratification. This variable not recorded in the NTDB. Scores and indices should be used as guides only. Physician judgment supersedes the model’s prediction. The TCRI should not stop a physician from working up symptoms or responding to a clinical risk assessment.

What is new?

We present a cardiac risk scoring system designed specifically for trauma. The TCRI includes some risk factors that are different from those commonly used in the peri-operative setting. Other risk factors that are common to both are weighted differently. Chest trauma is identified as a relatively minor risk factor for MI in the trauma setting. Our focus is on secondary rather than primary cardiac injury.

## Conclusions

This study confirms the overall low cardiac risk of the trauma population. Direct chest trauma is a risk factor for MI, but not the only one, nor even the strongest. Previous ischemic heart disease, comprising a history of MI and angina, represent the highest risk of trauma-associated MI. Thus, comorbidity, rather than the anatomic area of injury has the strongest association with MI. We propose that in the absence of symptoms, patients with a lower TCRI do not require any further cardiac screening or intensive cardiac monitoring. Patients with a higher score should undergo more intensive monitoring and cardiac risk reduction. Patients with symptoms that are indeterminate or difficult to evaluate should be considered for further workup based on a priori risk indicated by a higher TCRI.
